# Rapid LA-REIMS-based metabolic fingerprinting of serum discriminates aflatoxin-exposed from non-exposed pregnant women: a prospective cohort from the Butajira Nutrition, Mental Health, and Pregnancy (BUNMAP) Study in rural Ethiopia

**DOI:** 10.1007/s12550-024-00558-x

**Published:** 2024-09-11

**Authors:** Kokeb Tesfamariam, Vera Plekhova, Seifu H. Gebreyesus, Carl Lachat, Eugenio Alladio, Alemayehu Argaw, Bilal Shikur Endris, Meselech Roro, Sarah De Saeger, Lynn Vanhaecke, Marthe De Boevre

**Affiliations:** 1 Department of Food Technology, Safety, and Health, Faculty of Bioscience Engineering, Ghent University, Ghent, Belgium; 2 Department of Translational Physiology, Infectiology and Public Health, Laboratory of Integrative Metabolomics, Faculty of Veterinary Medicine, Ghent University, Merelbeke, Belgium; 3 Center of Excellence in Mycotoxicology and Public Health, Faculty of Pharmaceutical Sciences, Ghent University, MYTOX-SOUTH® Coordination Unit, Ghent, Belgium; 4 Department of Nutrition and Dietetics, School of Public Health, Addis Ababa University, Addis Ababa, Ethiopia; 5 Department of Biotechnology and Food Technology, Faculty of Science, University of Johannesburg, Doornfontein Campus, Gauteng, South Africa; 6 School of Biological Sciences, Queen’s University Belfast, Lisburn Road 97, Belfast, UK; 7 Department of Chemistry, University of Turin, Turin, Italy; 8 Department of Reproductive Health and Health Service Management, School of Public Health, Addis Ababa University, Addis Ababa, Ethiopia

**Keywords:** BUNMAP, Biomarkers, LA-REIMS, Metabolic features, Metabolomics

## Abstract

To date, the changes in maternal metabolic response associated with prenatal aflatoxin exposure remain largely unknown. This study investigated the effects of prenatal aflatoxin exposure on the maternal serum metabolome in rural Ethiopia. A total of 309 pregnant women were enrolled prospectively, and their serum aflatoxin concentrations were measured using targeted liquid chromatography coupled to tandem mass spectrometry (LC–MS/MS). Serum metabolic fingerprints were obtained using laser-assisted rapid evaporative ionization mass spectrometry (LA-REIMS), followed by combination of univariate and multivariate statistical modelling to evaluate changes in circulating metabolic features between aflatoxin-exposed and unexposed mothers and to select discriminatory metabolic features. The analysis revealed that 81.8% of women were exposed to aflatoxins, with a median concentration of 12.9 pg/mg albumin. The orthogonal partial least square discriminant analysis (OPLS-DA) regression model demonstrated significant disparities in the serum metabolome when comparing Ethiopian pregnant women with low vs high aflatoxin exposure. Thirty-two differentially expressed metabolic features were identified, affecting aminoacyl-tRNA biosynthesis pathway. Several discriminatory metabolites have been identified, including glutamine, tryptophan, tyrosine, carnosine, and 1-methylnicotinamide. In conclusion, our findings indicate that aflatoxin exposure during pregnancy have shown disparities in the maternal serum metabolome, primarily affecting protein synthesis. Further research is needed to identify specific metabolite biomarkers and elucidate the underlying mechanisms.

**Supplementary Information:**

The online version contains supplementary material available at 10.1007/s12550-024-00558-x.

## Introduction

Exposure to aflatoxins (toxic fungal secondary metabolites) is a global food safety and public health concern (Kumar et al. 2016). They frequently contaminate staple foods in low- and middle-income countries, and have been associated with a variety of adverse health outcomes, including liver cancer, immunosuppression, and impaired growth in animals and humans (Bennett 1987; Torres et al. 2015). Increased exposure to aflatoxin during pregnancy is of particular concern because exposure during this period can exert harmful effects on both mother and child (Kyei et al. 2020). Aflatoxins are transferred to the placenta via the umbilical cord blood and amniotic fluid, thus can adversely affect fetal growth and birth outcomes (Wild et al. 1991).

Pregnancy offers a window of opportunity to improve the future health of mothers and their children. During pregnancy, women undergo a number of physical and metabolic changes and adaptations to protect the developing fetus (Poon et al. 2018). Harmful environmental factors during pregnancy not only endanger the health of pregnant women but also affect the course of pregnancy and childbirth outcomes, posing a risk to fetal development and later life (Varshavsky et al. 2020). Therefore, prenatal exposure to widespread environmental toxins, such as aflatoxin, may hence lead to changes in metabolic pathways that may affect maternal health, fetal development, and health throughout life. These changes in metabolic pathways suggest potential effects of exposure, propose biomarkers of exposure, and lead to potential targets for intervention (Hasin et al. 2017; Eshelli et al. 2018; Doherty et al. 2022). Moreover, specific metabolic changes in biological fluids are often indicative of alterations in the individual’s physiological state, making them valuable markers of pathological conditions (Fiehn 2001).

Evaluation of the metabolic response enables describing the physiological responses or metabolic changes to environmental exposure (Buesen et al. 2017). Metabolomics studies have been widely used to investigate the effects of environmental exposures on maternal and fetal health, to better understand the pathogenesis of various diseases, and to identify biomarkers of disease (Cai et al. 2020). Metabolic fingerprinting is an untargeted metabolomics approach that aims to provide an unbiased metabolome assessment. In contrast, targeted metabolomics, also known as metabolic profiling, focuses on a priori defined molecules or specific chemical classes (Roberts et al. 2012). Ambient ionization mass spectrometry provides molecular information about the sample in near real-time without the need for prior sample preparation (Weston 2010). Laser Assisted–Rapid Evaporative Ionization Mass Spectrometry (LA-REIMS) is an ambient technique that allows rapid analysis of biological samples by directly ionizing them without the need for extensive sample preparation (Schäfer et al. 2009; Van Meulebroek et al. 2020).

Previously, we investigated the association between mycotoxin exposure during pregnancy and fetal growth trajectory and adverse birth outcomes in the same cohort of pregnant women (Tesfamariam et al. 2022). We reported that aflatoxin exposure was associated with poor fetal growth but not with birth outcomes. However, the possible physiological effects of aflatoxin exposure during pregnancy warrant further investigation. To date, the changes in maternal metabolic response associated with prenatal aflatoxin exposure remain largely unknown. The aim of the present study is to investigate the effects of aflatoxin exposure during pregnancy on the maternal serum metabolome in rural Ethiopia.

## Materials and methods

This study followed the STrengthening the Reporting of OBservational Studies in Epidemiology (STROBE) checklist (von Elm et al. 2014).

### Study design and participants

Data were collected from the Butajira Nutrition, Mental Health And Pregnancy (BUNMAP) prospective cohort study in Ethiopia, established at the Butajira Health and Demographic Surveillance Site. We collected samples from October 2017 to November 2020 from the Meskane and Mareko Districts in Ethiopia. The BUNMAP study protocol was approved by Addis Ababa University Institutional Review Board of (099/17/SPH). All women provided a written informed consent to participate.

We conducted a surveillance to identify and enroll pregnant women. Data collectors went door-to-door every month to check women’s pregnancy status using the WHO checklist (Secor and Stendig-Raskin 2018). After that, an ultrasound scan was conducted to confirm the pregnancy and estimate the gestational age. According to the WHO checklist, pregnant women were first invited to attend the nearby health centers for a baseline ultrasound and blood sampling. A total of 309 serum samples from pregnant women were analyzed using untargeted serum metabolomics.

Data and blood sample collection procedures were performed as previously described (Tesfamariam et al. 2022). Briefly, a phlebotomist collected a 5 mL of a venous blood sample from eligible women. A temporary field laboratory was set up in the study area where whole blood samples were collected, allowed to clot at room temperature, and then centrifuged to obtain serum. Then, the samples were then transported to the Ethiopian Public Health Institute and stored at − 40 °C. The samples were sent on dry ice to the Center of Excellence in Mycotoxicology and Public Health, Faculty of Pharmaceutical Sciences, Ghent University, Belgium, and stored at − 80 °C until analysis. Gestational age was determined by an experienced sonographer using a portable diagnostic imaging and full-color flow mapping ultrasound system (SonoSite M-Turbo, FUJIFILM SonoSite Inc., Bothell, WA 98021, USA) (Roro et al. 2019).

### Aflatoxinexposure determination

LC–MS/MS was utilized to analyze aflatoxin concentrations (as AFB_1_-lysine adduct (Renaud et al. 2022)) in blood serum as previously described (McCoy et al. 2005). Briefly, serum samples were thawed at 4 °C, vortexed, then 150 µL was transferred to a new polypropylene tube. Aliquots of each serum sample were digested overnight with pronase (Sigma Aldrich) at 37 °C to release protein-bound adducts. After centrifugation (4000 g, 10 min), 240 µL of the supernatant was evaporated to dryness under a gentle stream of N_2_ with a Turbovap (Biotage, LLC) at 40 °C for 15 min. The resulting residue was reconstituted by vortexing in 150 µL of the mobile phase injection solvent (H_2_O/MeOH, 80/20, v/v), filtered (0.22 μm, PVDF, Durapore®, Cork, Ireland), and transferred to HPLC vial with insert upon LC–MS/MS analysis. Sample analysis was performed on a Waters® Acquity UPLC system coupled to a XEVO TQ-S mass spectrometer (Waters®, Manchester, UK). All instrumental parameters were described in detail in a previous study (Tesfamariam et al. 2022). We used MassLynx™ version 4.1 and QuanLynx® version 4.1 (Waters®, Manchester, UK) software to process the data. The limit of detection (LOD) for AFB_1_-lysine was 0.03 ng/mL. An aliquot (50 µL) was taken from each blood sample to measure albumin levels (g/L) using a bromocresol green method albumin assay kit (MAK124) (Doumas et al. 1997). The serum AFB_1_-lysine level of each sample was adjusted according to its albumin content (i.e., pg AFB_1_-lysine/mg albumin). The aflatoxin classes were defined as aflatoxin-unexposed (AFB_1_-lysine < LOD) and exposed to aflatoxin (AFB_1_-lysine ≥ LOD).

### LA-REIMS untargeted serum metabolomics

LA-REIMS is an established platform for rapid biofluid fingerprinting, and details of the analytical procedure have been reported previously (Plekhova et al. 2021). Briefly, the system consists of a Xevo G2- XS QToF mass spectrometer equipped with the REIMS source (Waters, Manchester, UK), a Harvard 11 Elite syringe pump (Harvard Apparatus, USA) serving as the solution delivery system, an Opolette 2940 mid-IR laser system (OPOTEK, USA) pumped by Nd:YAG laser and energy output controlled with an optical parametric oscillator fixed at 2940 nm. The laser beam is focused on the sample surface using a set of metal-coated mirrors and a plano-convex lens (Thorlabs, USA). A custom-designed motorized xy-precision position stage serves as a sample surface and provides automated analysis. For analysis, 100 µL of each participant and untreated serum sample was placed in random order in 96-well plates. Each sample was ablated for 3 s (laser Q-switch delay time 165 µs) with 15-s cool down time between the samples. The resulting sample vapor was collected using a PTFE tube (2.5 m, 3.2-mm outer diameter (OD), 1.6-mm inner diameter (ID), Sigma Aldrich) and transported to the level of the source. Within the stainless-steel T-piece, the sample vapor was mixed with the solvent matrix (LC–MS grade 2-propanol, Sigma Aldrich) that was infused continuously through the analysis at a constant rate of 0.20 mL/min. After that, the sample/solvent mixture was then transported to the MS system for further ionization, mass separation, and detection, with the cone voltage set at 30 V, the heater bias voltage set at 40 V, and the scan time set at 0.7 s. The signal was captured using MassLynx™ version 4.1 software. A set of pooled quality control samples was analyzed after every 20 biological samples to assess the quality of the data obtained. The signal from leucine-enkephalin (Sigma Aldrich), spiked in the solvent matrix at a concentration of 10 ng/mL, acted as internal standard (ISTD) and was monitored throughout the analysis to assess instrumental stability and used for lock-mass correction. The ensure mass precision, the MS system was calibrated prior to the analysis according to the manufacturer’s instructions.

To compensate for the batch differences and instrumental signal drift during analysis, the intensities acquired raw features’ were normalised based on the total signal intensity/total ion current (TIC) of each sample and LOESS (locally weighted smoothing) based on quality control (QC) samples (Di Guida et al. 2016). This combination was selected based on the ability of various data normalization strategies and their combinations (TIC, LOESS, median intensities in QC samples, ISTD intensity and no normalization) to achieve QC sample clustering on the PCA-X model score plot and variable distribution normality (skewness and kurtosis) after logarithmic transformation and Pareto-scaling.

### Statistical analysis

The mean ± (standard deviation [SD]) for continuous variables and number (percent) for categorical variables were used to express the data. Univariate analysis was applied to identify the features with significantly different means in the aflatoxin-exposed and non-exposed groups, based on *t*-test and the Benjamini–Hochberg method to control the false discovery rate (FDR) adjusted *P* < 0.05 and fold change (FC > 2) (Benjamini and Hochberg 1995). To ensure normality of the distribution and the equality of the variance, the data were log-transformed and pareto scaled before univariate testing. Unsupervised and supervised multivariate analyses using principal component analysis (PCA) and orthogonal partial least square discriminant analysis (OPLS-DA) were conducted to account for possible metabolite interactions. First, PCA was carried out to visualize general trends in the sample distribution and identify outliers based on the 95% Hotelling’s T2 criterion. Then, OPLS-DA model was used to evaluate driving forces between detected metabolome changes in aflatoxin exposed and unexposed groups. Validity of the OPLS-DA model was assessed by permutation testing (*n* = 100), cross-validated analysis of variance (*P* < 0.05) and by inspection of R^2^Y (goodness-of-fit) and Q^2^Y (goodness-of-prediction, based on cross-validation with seven segments) model characteristics (Eriksson et al. 2008; Triba et al. 2015). The metabolic features contributing the most to the difference between investigated groups were selected based on the VIP score > 1 and *P* < 0.05 obtained by the OPLS-DA model. Pathway enrichment analyses was conducted using mummichog algorithm, to gain functional insight directly from *m/z* features bypassing metabolite identification (Li et al. 2013). Statistical analysis was carried out using R language (version 4.2.0), SIMCA (version 17.0, Sartorius, Umea, Sweden), and MetaboAnalyst 5.0 analytical platform (Pang et al. 2021). Within the R environment, the ‘ropls’ (Version 3.17) package was used for data analysis http://bioconductor.org/packages/release/bioc/html/ropls.html) (Thévenot et al. 2015).

## Results

### Baseline characteristics of the study participants

**Fig. 1:**
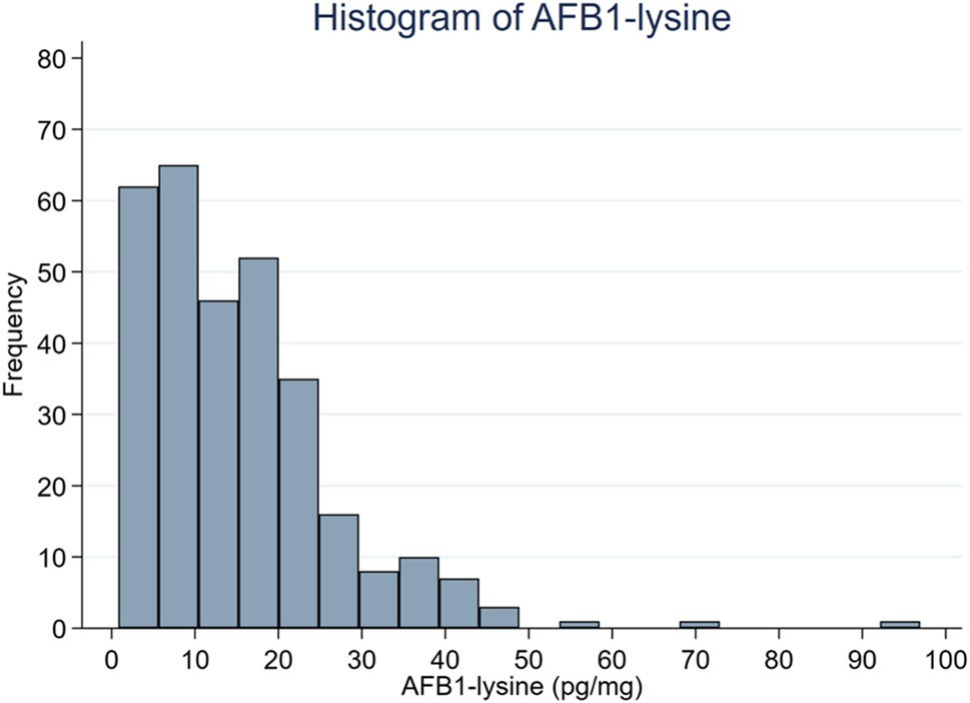
Histogram of the AFB1-lysine adduct levels

The baseline characteristics of the study participants are summarized by study groups in Table 1. Overall, baseline maternal socio-demographic characteristics were comparable between aflatoxin-exposed and unexposed groups. The mean (SD) maternal body mass index of the women at enrollment was 21.6 (2.87) kg/m^2^ and gestational age was 19.1 (3.71) weeks. About 46% of the mothers were from food insecure households and 12.9% were underweight. Of the 309 pregnant women enrolled in the study, 253 (81.8%) had detectable levels of AFB_1_-lysine adducts and were classified as exposed to aflatoxin. The descriptive statistics of AFB1-lysine (pg/mg) are presented in Table 2, while Fig. 1 illustrates the data’s distribution.

**Table 1:** Baseline characteristics of study participants by aflatoxin exposure status^1^

Maternal characteristics	Total (*n* = 309)	Unexposed (*n* = 56)	Exposed (*n* = 253)
Fetal sex, female	138 (47.6)	26 (49.1)	112 (47.3)
Maternal age, years	25.7 ± 4.46	25.7 ± 4.00	25.7 ± 4.60
Body mass index (kg/m^2^)			
Underweight (< 18.5)	40 (12.9)	11 (19.6)	29 (11.5)
Normal weight (18.5–25)	245 (79.3)	43 (76.8)	202 (79.8)
Overweight (25–30)	18 (5.80)	2 (3.57)	16 (6.32)
Obese (> 30)	6 (1.94)	-	6 (2.37)
Maternal education			
No formal education	247 (79.9)	39 (69.6)	208 (82.2)
Primary education	57 (18.4)	15 (26.8)	42 (16.6)
Secondary and above	5 (1.70)	2 (3.60)	3 (1.20)
Household wealth tertiles			
Lowest	93 (30.1)	17 (30.4)	76 (30.0)
Middle	106 (34.3)	20 (35.7)	86 (34.0)
Highest	110 (35.6)	19 (33.9)	91 (36.0)
Household food security^2^			
Food secure	166 (54.0)	30 (53.6)	136 (54.2)
Mildly food insecure	30 (9.77)	7 (12.5)	23 (9.20)
Moderately food insecure	99 (32.3)	18 (32.1)	81 (32.3)
Severely food insecure	12 (3.90)	1 (1.80)	11 (4.40)
Depression status of the mother			
Not depressed	222 (72.3)	39 (69.6)	183 (72.9)
Depressed	85 (27.7)	17 (30.4)	68 (27.1)
Agro-ecological zones			
Lowland	134 (43.4)	19 (33.9)	115 (45.5)
Midland	97 (31.4)	20 (35.7)	77 (30.4)
Highland	78 (25.2)	17 (30.4)	61 (24.1)
Mid-upper arm circumference, cm	24.8 ± 2.34	25.2 ± 2.20	24.7 ± 2.40
Hemoglobin level, g/dL	13.0 ± 1.40	13.3 ± 1.60	13.0 ± 1.40

^1^Values are mean ± SD or *n* (%)

^2^Assessed using FANTA/USAID’s Household Food Insecurity Access Scale

The aflatoxin categories were defined as follows: unexposed to AFB_1_-lysine ( < LOD) and exposed to AFB_1_-lysine (≥ LOD). *LOD*, Limit of detection; *SD*, standard deviation

**Table 2:** Summary of descriptive statistics of AFB1-lysine adduct levels

AFB1‐lysine (pg/mg)	
Mean	15.2
Standard deviation	12.2
Median	12.9
Q1	7.9
Q3	20.9

Abbreviations: *AFB1*, aflatoxin B1; *Q1*, first quartile; *Q3*, third quartile

### Univariate analysis of LA-REIMS metabolic features

**Fig. 2:**
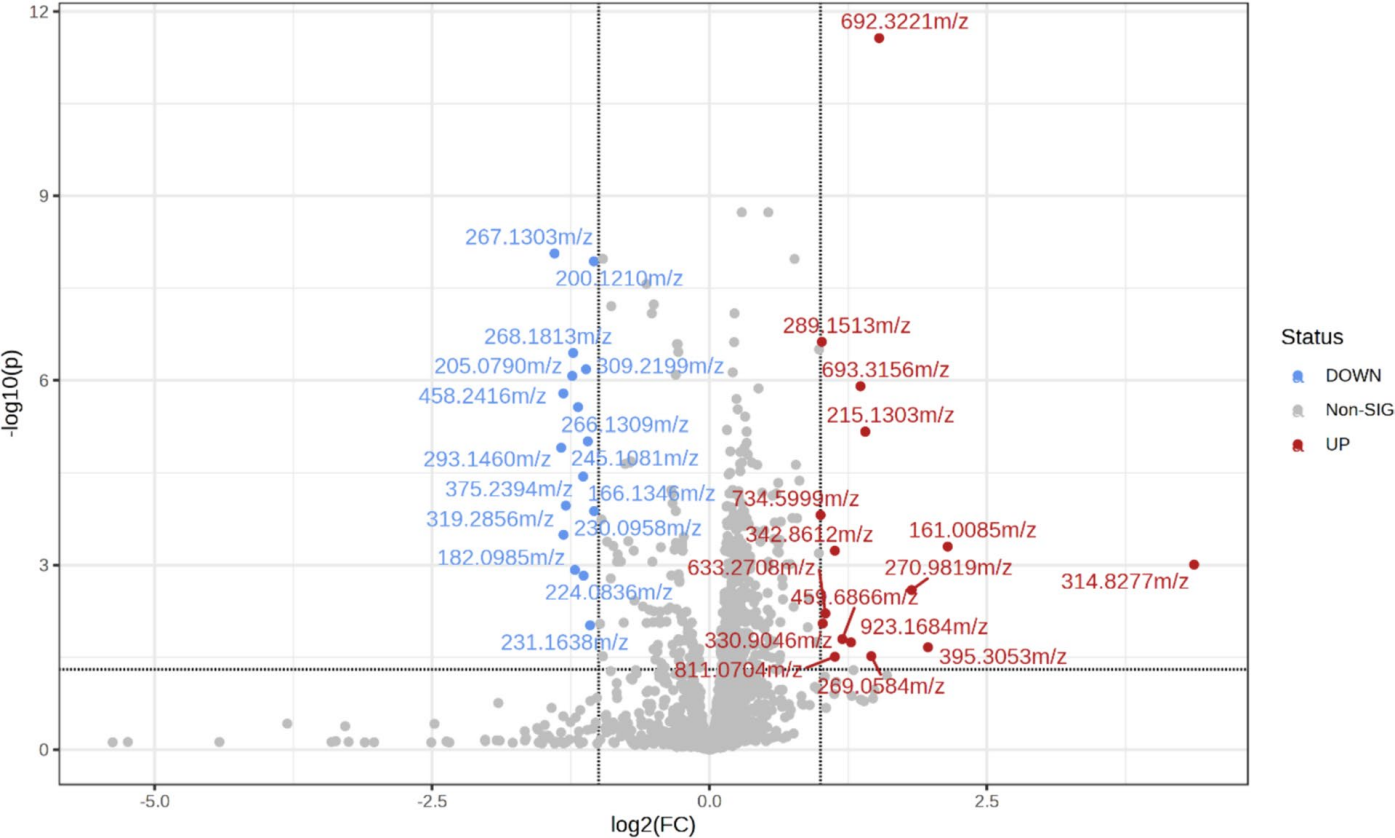
Volcano plot of features acquired in the negative ionization mode. Threshold for the significance of features is set at fold change (FC) > 2 and false discovery rate (FDR) adjusted *P* < 0.05 between investigated groups. Red circles represent significantly upregulated and blue circles downregulated features in the aflatoxin-exposed group. The label of significant features indicates their accurate *m/z* (mass-to-charge) values

Untargeted metabolomics from maternal serum samples identified a total of 1957 metabolic features. Among these features investigated, 32 showed a significant difference between aflatoxin-exposed and unexposed groups based on FC > 2 and FDR-corrected *P* < 0.05 (Supplementary Table S1). Of these metabolic features, 16 features were upregulated, and 16 features were downregulated in the aflatoxin-exposed group (Fig. 2).

### Multivariate analysis of metabolic fingerprints

**Fig. 3:**
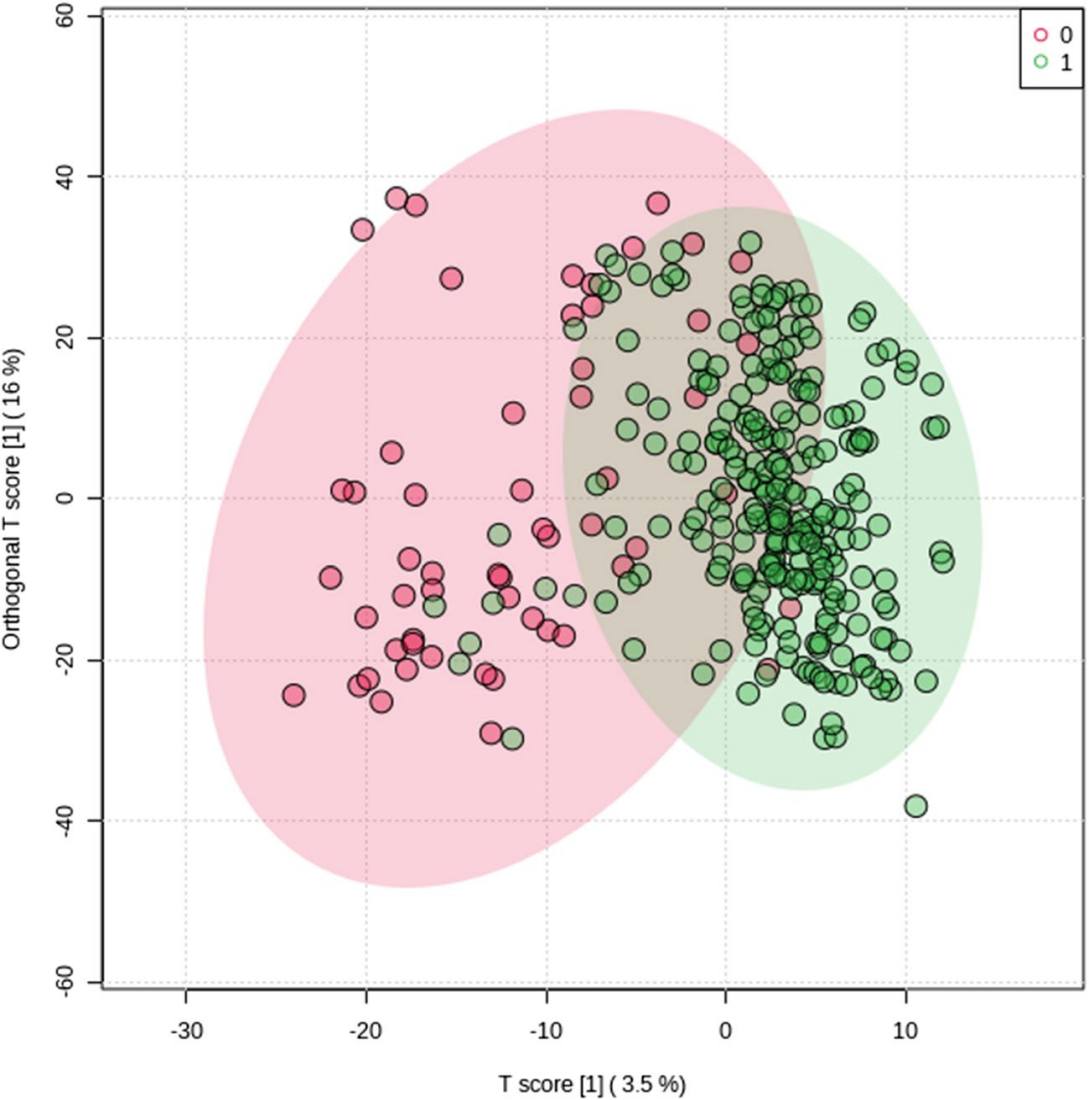
OPLS-DA score plot constructed based on the validated model (R^2^Y = 0.877 and Q^2^Y = 0.601) constructed from acquired serum metabolome (green – exposed to aflatoxin, red – unexposed to aflatoxin)

Principal component analysis (PCA) was applied to evaluate natural sample clustering and separation between the exposed and unexposed groups and to identify outlying observations. The first principal component (PC1) explained 55.9% of the total variance and PC2 explained 17.2% of the variance, with the maximum total variance explained by five PCs being 91.8% (Supplementary Fig. S1). The OPLS-DA model was constructed to evaluate the ability to discriminate between aflatoxin-exposed and unexposed women based on their serum metabolic fingerprint as well as identify differential metabolic features characteristic of aflatoxin exposure. The OPLS-DA score plot of the metabolic features clearly illustrated the grouping of participants into two separate clusters according to aflatoxin exposure. Validity of the OPLS-DA model was confirmed by CV-ANOVA (*P* < 0.05), permutation testing (*n* = 100), and model diagnostics (i.e., R^2^*Y* = 0.877 and Q^2^*Y* = 0.601, calculated by sevenfold cross-validation) (Fig. 3 and Supplementary Fig. S2). Additionally, the exposure level was categorized as low exposure for those with aflatoxin concentrations below the median, and as high exposure for those with concentrations at or above the median. The OPLS-DA score plot (Supplementary Fig. S4) also demonstrates group separation between low and high aflatoxin exposure.

**Fig. 4:**
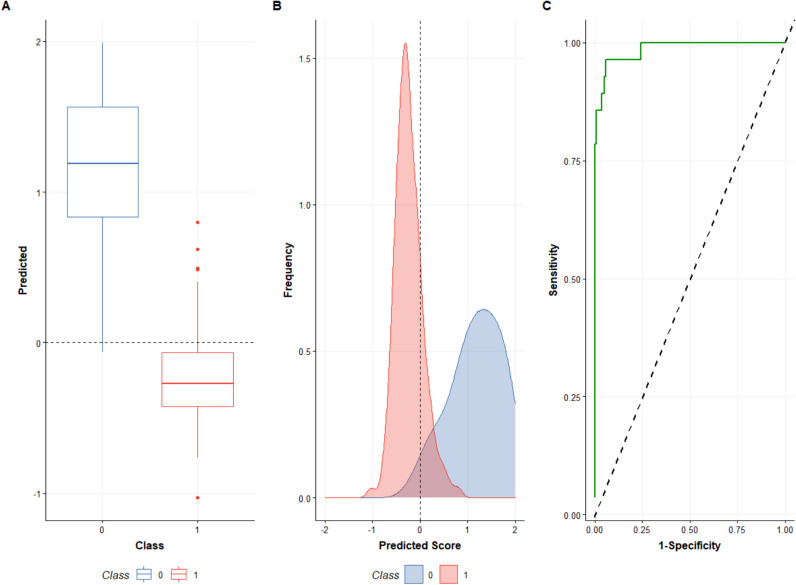
(**A**) Box plot showing the distribution of known exposed and unexposed participant’s data in both training and test sets, and the class cut-off (dotted line); (**B**) Probability density functions for positive (exposed) and negative (unexposed) classes in the training and test sets; (**C**) Receiver Operating Characteristic curve of the OPLS-DA model performance. The area under the curve (AUC) is 0.93, which is close to the maximum possible value of 1

The Area Under the Receiver Operating Characteristic (AU-ROC) curve for the validated OPLS-DA model for classifying pregnant women by aflatoxin exposure reached a value of 0.93. These results indicate that our serum metabolome-based model can effectively discriminate between women who are exposed to aflatoxin and those who are not and has both sufficient sensitivity and specificity as a classifier of aflatoxin exposure during pregnancy (Fig. 4).

Selection of the features contributing most to the discrimination between aflatoxin exposed and unexposed groups was based on VIP score > 1 and *P* < 0.05 from OPLS-DA analysis. Applying these criteria, we identified 601 distinct features from the exposed and unexposed groups that can act as potential metabolic markers of aflatoxin exposure. The top 16 features identified by highest VIP value are presented in Supplementary Fig. S3.

### Mummichog pathway analysis

**Fig. 5:**
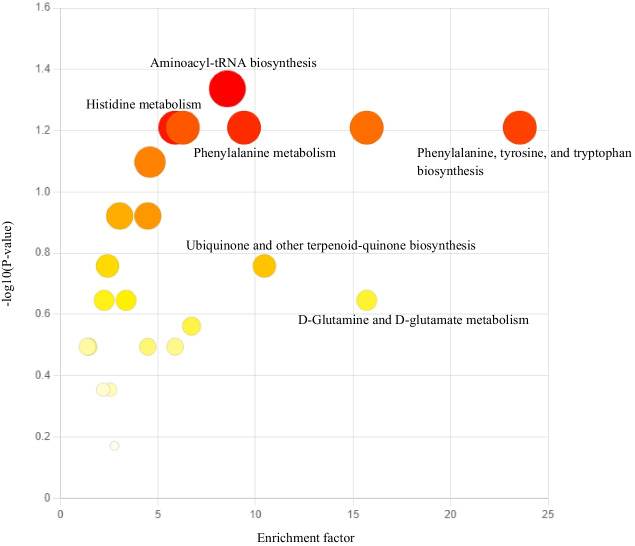
Metabolic pathway enrichment plot. The identified pathways are ordered by corresponding enrichment factor (x-axis) and negative logarithm (base 10) of the *P*-value (y-axis). The enrichment factor of a pathway is calculated as the ratio between the number of significant pathway hits and the expected number of compound hits within the pathway

To further investigate the biological significance of these findings, we performed a pathway analysis using mummichog algorithm, incorporating preselected features from the OPLS-DA model. The results of the pathway analysis are presented in Fig. 5. Several putative identities reached significance in the permutation tests (*P* < 0.05), namely: glutamine (KEGG ID C00064), tryptophan (KEGG ID C00078), tyrosine (KEGG ID C00082), carnosine (KEGG ID C00386) and 1-methylnicotinamide (KEGG ID C02918). The pathway analysis revealed that several amino acid metabolism pathways were indeed enriched, including aminoacyl-tRNA biosynthesis (C00064, C00078, C00082), histidine metabolism (C00386), phenylalanine metabolism (C00082), phenylalanine, tyrosine and tryptophan biosynthesis (C00082), and nicotinate and nicotinamide metabolic pathways (C02918). However, only alterations in the aminoacyl-tRNA biosynthesis pathway reached statistical significance (*P* = 0.04).

## Discussion

Even though aflatoxin exposure has been linked to numerous adverse health effects, including fetal growth restriction, immunomodulation, and growth retardation in low-income countries (Gong et al. 2016; Tesfamariam et al. 2020; Ismail et al. 2021), metabolic disturbances associated with prenatal aflatoxin exposure are poorly understood. Here, we present findings of an untargeted metabolomics analysis of maternal serum samples to explore the relationship between aflatoxin exposure and metabolic signatures during the first and second trimesters of pregnancy in rural Ethiopia. To the best of our knowledge, the present study is the first to investigate potential differences in maternal serum metabolic signatures based on their aflatoxin exposure status during pregnancy in rural Ethiopia.

Measuring more than 1957 metabolic features in serum samples using LA-REIMS and comparing metabolic features according to exposure, our analyses showed clear discrimination between the metabolome of aflatoxin-exposed and unexposed women. Furthermore, we identified 32 significantly differentially expressed metabolic features between the two groups, with 16 upregulated and 16 downregulated in the aflatoxin-exposed group. Although specific metabolite identification in the LA-REIMS is challenging, some putative identities could be achieved through mummichog pathway analysis. For the other unknown compounds, the mass distribution of the proposed biomarkers provides valuable insights into their possible identities and underlying metabolic processes.

The majority of the putative biomarkers were found in the low molecular mass region (100–350 Da), suggesting they might be amino acids and fatty acids. These molecules function as intermediates in various metabolic pathways. This is supported by the results of our pathway analysis that aflatoxin causes disruptions of amino acid metabolism and by several putatively identified amino acids (i.e., glutamine, tryptophan, tyrosine and carnosine) among the compounds contributing the most to the differentiation according to aflatoxin exposure based on the valid OPLS-DA model. The results were consistent with the previous findings of an animal study that aflatoxin induced a significant increase in the levels of amino acids (Zhang et al. 2011). In contrast, the proposed few higher molecular weight biomarkers (600–900 Da) are likely representatives of more complex lipids such as phosphatidylcholines (St John et al. 2017). These molecules have a role in more complex metabolic pathways such as DNA replication and transcription, protein synthesis, and membrane biogenesis. While the proposed metabolomic features may serve as potential biomarkers, their molecular structure and metabolite identity should be verified by fragmentation experiments or comparison to commercial or synthesized metabolite standards (Johnson et al. 2016). Furthermore, additional validation in separate prospective cohorts is required to establish the robustness of these features.

Furthermore, our findings suggest that aflatoxin exposure during pregnancy significantly altered the aminoacyl-tRNA biosynthesis pathway. The aminoacyl-tRNA biosynthesis pathway is a process in which amino acids are bound to the corresponding tRNA molecules. This process is essential for protein synthesis and is regulated by aminoacyl-tRNA synthetases (Nie et al. 2019). Previous studies also confirm the suppressive effect of aflatoxin exposure on protein synthesis, resulting in stunted growth in children and some in vitro and animal models (Williams et al. 2004). Similar effects can be expected regarding fetal growth and development during pregnancy. Specifically for gut intestinal cell culture, combined transcriptome and proteome analysis also confirms that the toxic effect of aflatoxin exposure resulting in low cell viability and stunted growth due to reduced protein and miRNA synthesis (Gao et al. 2022).

Moreover, aflatoxin-induced oxidative stress (Da Silva et al. 2018), resulting from an imbalance between reactive oxygen species and antioxidant defense mechanisms, may also affect the aminoacyl-tRNA biosynthesis pathway and other components of cellular machinery. Indeed, aflatoxin contamination of the feed has been linked to changes in amino acid profiles and impairment of the antioxidant system in an experimental porcine model (Duan et al. 2014). Interestingly, supplementation of glutamine, one of the amino acids found to be significantly affected by aflatoxin exposure in our study, mitigated the effects of toxin-contaminated feed to some extent. The latter supports the role of glutamate in the pathogenesis of aflatoxin exposure. In addition, animal studies have demonstrated the inhibitory effect of aflatoxin specifically on aminoacyl-tRNA synthetases, supporting our findings (Wagner and Unterreiner 1981; Zhang et al. 2011).

It is worth noting that aflatoxin exposure may not be the sole source of the observed metabolic shifts as other factors, such as diet composition and quality, co-exposure to other environmental and mycotoxins and other exposome components also impact the overall metabolism of pregnant women (Watson et al. 2016) and the distribution of these confounders within our study groups are unknown. Additionally, external environmental influences during pregnancy may cause fluctuations in metabolomic profiles (Monni et al. 2021). One of the limitations of our study is the absence of specific data on nutrient values. This limitation means that potential influences of micronutrient deficiencies on the serum metabolome could not be directly assessed. Future research should include comprehensive nutritional assessments to elucidate the interactions between nutritional status and aflatoxin exposure on metabolic outcomes. However, measuring the exposome, which refers to the comprehensive assessment of environmental exposures an individual experiences throughout their lifetime, is challenging (Sillé, 2020). Future research may propose to confirm and expand on our findings by incorporating other omics data, such as transcriptomics, proteomics, and epigenomics, which offer a more comprehensive understanding of the biological mechanisms underlying aflatoxin exposure and its health effects (Cai et al. 2020). Therefore, a thorough understanding of the intricate relationships between aflatoxin exposure, maternal metabolism, and fetal development requires extensive studies that take into account a variety of exposure sources, significant covariates, and the monitoring of metabolomic changes over time.

While the untargeted metabolomics method is valuable for analyzing metabolites in a sample, it has a drawback regarding the uncertainty surrounding the identities of the acquired metabolite features. In the traditional metabolomics workflow, which is hyphenated between chromatography and mass spectrometry, identification of the compounds is based on the accurate mass, molecule fragmentation pattern (MS/MS), comparison to the pure analytical standards and the retention time on the chromatographic column (Hissong et al. 2022). Nevertheless, accurate mass measurements are the only basis for metabolite characterization by LA-REIMS, which has significant implications for metabolite identification and to some extent absolute quantitation. The structural identification of features is hampered by the absence of chromatographic separation. In our study, putative identification of several amino acids involved in aflatoxin pathogenesis was achieved by direct mapping of detected features to known metabolic pathways (i.e., mummichog algorithm). However, these identified molecules represent only a very limited portion of the captured metabolome. Future research focusing on the in-depth characterization of the maternal serum metabolome using hyphenated techniques (e.g., LC–MS /MS) is recommended to determine the identity of the remaining discriminating metabolites. However, it should be noted that the rapid and direct metabotyping enabled by the LA-REIMS platform, which allowed the analysis of a large number of serum samples, as well as the results obtained in identifying general shifts in metabolic pathways and major discriminatory metabolites, demonstrate the validity of LA-REIMS as an excellent prescreening platform for future, more costly and laborious identification experiments.

In conclusion, our findings indicate that aflatoxin exposure during pregnancy demonstrated significant disparities in the maternal serum metabolome. These changes, potentially linked to modest concentrations of serum adducts, may contribute to adverse maternal and fetal outcomes associated with aflatoxin exposure during pregnancy. Furthermore, if the metabolic changes by aflatoxin exposure persist, they may lead to long-term sequelae that extend into adulthood. Further research is needed to identify the specific metabolite biomarkers and to elucidate the mechanisms by which these changes occur. Nonetheless, our results underscore the importance of reducing aflatoxin exposure during pregnancy in rural Ethiopia to improve maternal and fetal health outcomes.

## Supplementary Information

Below is the link to the electronic supplementary material.

Supplementary file1 (DOCX 351 KB)

Supplementary file2 (CSV 5 KB)

## Data Availability

Anonymized data will be made available upon reasonable request pending review by the principal investigator and the signing of the data-sharing agreement.
