# Supplementary material for: Rapid LA-REIMS-based metabolic fingerprinting of serum discriminates aflatoxin-exposed from non-exposed pregnant women: a prospective cohort from the Butajira Nutrition, Mental Health, and Pregnancy (BUNMAP) Study in rural Ethiopia
Source: Mycotoxin Res. 2024 Sep 11;40(4):681–91. doi: 10.1007/s12550-024-00558-x (PMC11480126; doi:10.1007/s12550-024-00558-x)
Supplement: Supplementary file 1 — Supplementary file1 (DOCX 351 KB) [file 12550_2024_558_MOESM1_ESM.docx]

**Supplementary information**

**Supplemental Table S1.** Metabolic features that showed a significant difference between aflatoxin-exposed and unexposed groups based on FC >2 and FDR-corrected *P* < 0.05

| **Features** | **FC** | **log2(FC)** | ***P*.ajusted** | **-LOG10(p)** |
| --- | --- | --- | --- | --- |
| 692.3221m/z | 2.8854 | 1.5287 | 2.71E-12 | 11.567 |
| 267.1303m/z | 0.37985 | -1.3965 | 8.64E-09 | 8.0633 |
| 200.1210m/z | 0.48554 | -1.0423 | 1.16E-08 | 7.9357 |
| 289.1513m/z | 2.0168 | 1.0121 | 2.37E-07 | 6.6259 |
| 268.1813m/z | 0.42672 | -1.2286 | 3.59E-07 | 6.4448 |
| 309.2199m/z | 0.46243 | -1.1127 | 6.61E-07 | 6.1795 |
| 205.0790m/z | 0.42429 | -1.2369 | 8.48E-07 | 6.0718 |
| 693.3156m/z | 2.5678 | 1.3605 | 1.25E-06 | 5.9036 |
| 458.2416m/z | 0.4014 | -1.3169 | 1.64E-06 | 5.786 |
| 266.1309m/z | 0.43999 | -1.1845 | 2.73E-06 | 5.5645 |
| 215.1303m/z | 2.6456 | 1.4036 | 6.80E-06 | 5.1677 |
| 245.1081m/z | 0.46756 | -1.0968 | 9.70E-06 | 5.0134 |
| 293.1460m/z | 0.396 | -1.3364 | 1.23E-05 | 4.9092 |
| 166.1346m/z | 0.45422 | -1.1386 | 3.63E-05 | 4.44 |
| 375.2394m/z | 0.40754 | -1.295 | 0.0001077 | 3.9678 |
| 230.0958m/z | 0.48638 | -1.0398 | 0.00013249 | 3.8778 |
| 734.5999m/z | 2.0008 | 1.0006 | 0.00015336 | 3.8143 |
| 319.2856m/z | 0.40172 | -1.3157 | 0.00032088 | 3.4937 |
| 161.0085m/z | 4.4262 | 2.1461 | 0.00049977 | 3.3012 |
| 342.8612m/z | 2.1875 | 1.1293 | 0.00058292 | 3.2344 |
| 314.8277m/z | 20.632 | 4.3668 | 0.0009822 | 3.0078 |
| 182.0985m/z | 0.43148 | -1.2126 | 0.0011974 | 2.9217 |
| 224.0836m/z | 0.45548 | -1.1345 | 0.0014856 | 2.8281 |
| 270.9819m/z | 3.534 | 1.8213 | 0.0025482 | 2.5938 |
| 633.2708m/z | 2.0629 | 1.0447 | 0.0061187 | 2.2133 |
| 330.9046m/z | 2.0286 | 1.0205 | 0.0089118 | 2.05 |
| 231.1638m/z | 0.47422 | -1.0764 | 0.0095485 | 2.0201 |
| 459.6866m/z | 2.2925 | 1.1969 | 0.015992 | 1.7961 |
| 923.1684m/z | 2.4201 | 1.2751 | 0.018087 | 1.7426 |
| 395.3053m/z | 3.9141 | 1.9687 | 0.02171 | 1.6633 |
| 269.0584m/z | 2.7462 | 1.4574 | 0.03029 | 1.5187 |
| 811.0704m/z | 2.1881 | 1.1297 | 0.030929 | 1.5096 |


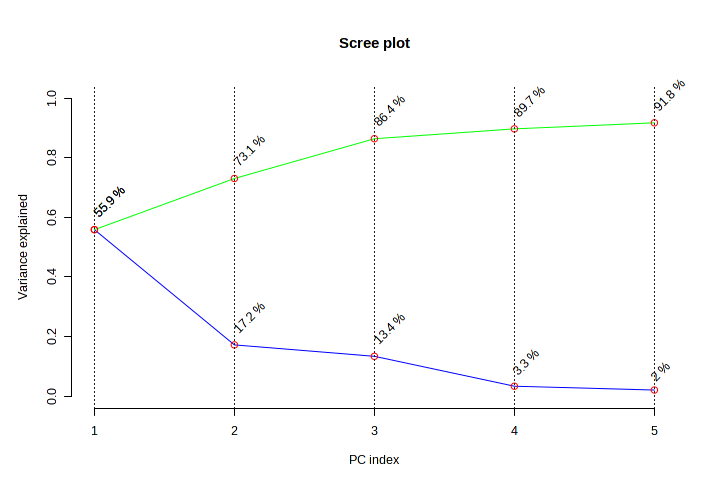


**Supplemental Figure S1.** Scree plot shows the variance explained by principal components (PCs). The green line on top shows the accumulated variance explained; the blue line underneath shows the variance explained by individual PC


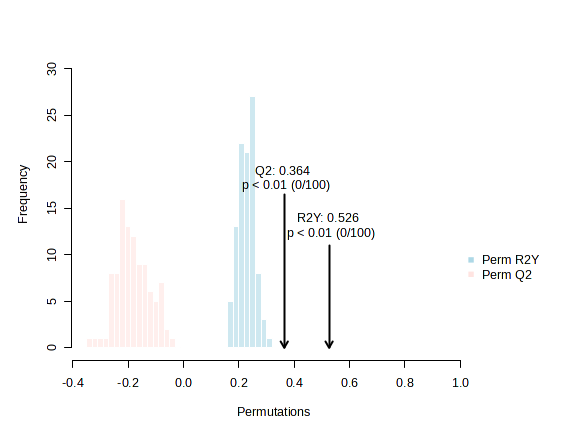


**Supplemental Figure S2.** Permutation analysis, showing the observed and cross-validated R^2^Y and Q^2^ values. All models based on the randomly permuted dataset have lower R^2^Y and Q^2^ values than the original model. This concludes that the goodness of fit and predictive properties of the original model are not the result of a random distribution/event and are statistically valid


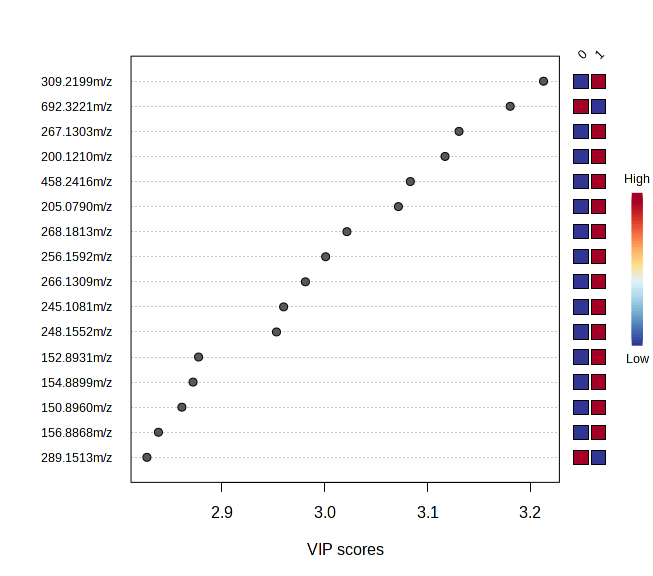


**Supplemental Figure S3**. Features responsible for the observed segregation between aflatoxin exposed and unexposed groups as filtered out by OPLS-DA (top 16 metabolite features). The colored boxes on the right indicate relative intensity (blue – lower, red – higher) of the corresponding metabolite in each group under study (0 – unexposed to aflatoxin, 1 – exposed to aflatoxin)


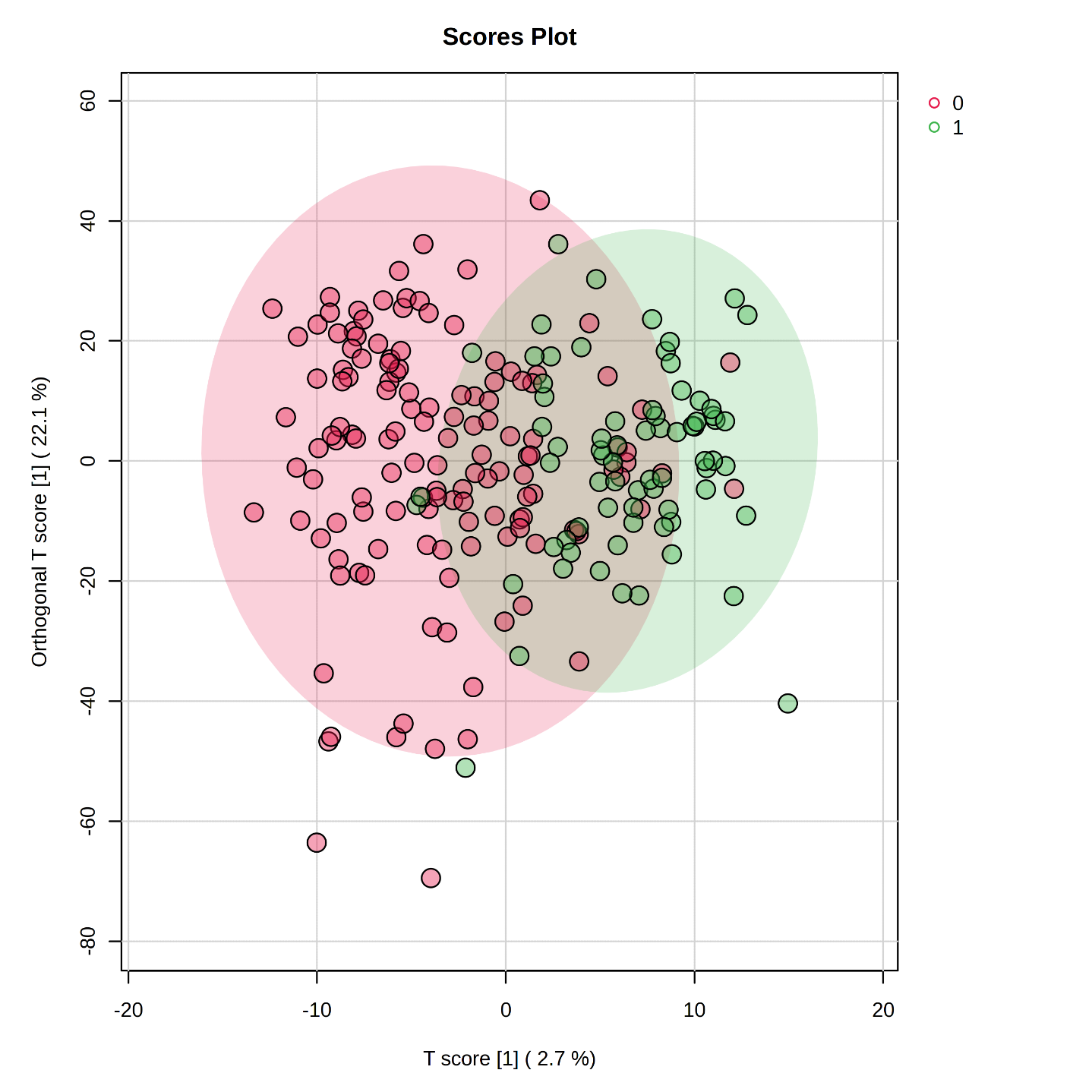


**Supplemental Figure S4.** OPLS-DA score plot constructed from acquired serum metabolome (1 – above median exposure to aflatoxin, 0 – below median exposure to aflatoxin)
